# A Comparison of Machine versus Free-Weight Squats for the Enhancement of Lower-Body Power, Speed, and Change-of-Direction Ability during an Initial Training Phase of Recreationally-Active Women

**DOI:** 10.3390/sports7100215

**Published:** 2019-09-30

**Authors:** Neil A. Schwarz, Sean P. Harper, Andy Waldhelm, Sarah K. McKinley-Barnard, Shelley L. Holden, John E. Kovaleski

**Affiliations:** 1 Department of Health, Kinesiology, and Sport, University of South Alabama, Mobile, AL 36688, USA; sh1721@jagmail.southalabama.edu (S.P.H.); sarahmckinley@southalabama.edu (S.K.M.-B.); sholden@southalabama.edu (S.L.H.); jkovales@southalabama.edu (J.E.K.); 2 Department of Physical Therapy, University of South Alabama, Mobile, AL 36688, USA; awaldhelm@southalabama.edu

**Keywords:** periodization, squat mode, athletic performance, strength and conditioning, females, strength training, plyometrics

## Abstract

The purpose of this study was to examine differences between a free-weight squat (FWS) and machine squat (MS) during an initial resistance training phase for augmentation of performance tests in recreationally active women. Twenty-seven women (22.7 ± 3.5 years) were block-randomized to three groups: FWS, MS, or control (CON) and completed pre- and post-testing sessions consisting of the squat one-repetition maximum (1-RM), vertical jump, pro-agility test, zig-zag change-of-direction (COD) test, and 30-meter sprint. Participants trained two sessions per week for six weeks by performing jumping, sprinting, and COD drills followed by FWS, MS, or no squats (CON). Peak jump power increased for CON (*p* = 0.03) and MS (*p* < 0.01) groups. Change in peak jump power was greater for the MS group compared with the FWS group (*p* = 0.05). Average jump power increased for the MS group (*p* < 0.01). Change in average jump power was greater for the MS group compared with the CON group (*p* = 0.04). Vertical jump height, pro-agility, 30-meter sprint, and zig-zag COD tests improved over time (*p* < 0.01), with no difference between groups (*p* > 0.05). Machine squat training maximized jumping power compared with FWS training and CON. Both resistance training groups and the CON group improved equally in the pro-agility, 30-meter sprint, and zig-zag COD tests. Machine squat training may provide performance-enhancing benefits of equal or superior value to those obtained with free-weight squat training in recreationally active women during an initial training mesocycle. These findings also stress the importance of task-specific training in this population of untrained women, as the control group improved in terms of performance to the same degree as both resistance training groups.

## 1. Introduction

Resistance training is believed to be important for enhancing an athlete’s potential performance [1]. A resistance training program that mimics the specific movements and physiological stresses of a given sport may enhance performance in that sport [2]. Specifically, the squat movement pattern is arguably one of the most fundamental movements necessary to improve sport performance, to reduce injury risk, and to support lifelong physical activity, primarily because of its recruitment of a large portion of lower-extremity musculature [3]. For instance, in soccer players, Chelly et al. [4] and Styles et al. [5] demonstrated that free-weight squat training increases the lower-body power output, sprint times, and vertical jump performance. Likewise, increases in maximal squat strength improve sprint performances in rugby league players [6].

The squat exercise contains a multitude of variations, including squats using free weights, i.e., a barbell with weight plates and machine variations of the squat, one of the most popular being the hack squat machine. Additionally, resistance training machines, such as the leg press, are also popular choices for resistance training of the lower body. In many resistance training programs, a combination of different squat variations and machines are used concurrently to supplement each other and increase training volume. A published account of “The Science of Strength” suggests free-weight exercise is established as undoubtedly eliciting superior transfer to sports performance activities when compared with machine exercise [1]. However, others contend that the superiority of free-weight exercise for sports-specific tasks is not evidence-based [7]. At best, the comparison between free-weight and machine exercise for improving performance has produced conflicting results. In support of machine exercise being as effective as free weights, Silvester and Bryce [8] studied the effects of free-weight and machine resistance training on jumping ability and lower-body strength following an 11-week program in untrained college-aged men. The authors reported that vertical jump height had a similar significant increase for both the Universal machine and free-weight training groups. The investigators concluded that variable resistance and traditional free weights produced similar effects on jumping ability and strength. Likewise, Rossi et al. [9] conducted a study to compare the effectiveness of either barbell squat, leg press, or a combination of both, on multiple functional outcomes. The pre–post test results showed significant increases in fat-free mass, balance test performance, vertical jump height, and 1-RM leg press, with no significant differences among the groups for any of the outcome measures. Rossi et al. [9] concluded that the three training protocols had similar significant positive effects on the vertical jump and balance tests. Conversely, other studies have shown machine training to be inferior to free weights for performance. In a study utilizing periodized, machine-based squat training [10], researchers reported the training to be ineffective in eliciting improved functional performance in untrained men and women when compared with control subjects. More recently, Wirth et al. [11] studied the effects of squat and leg-press training on jump performance and strength measurements in athletes. Results showed that the free-weight squat group significantly increased vertical jump performance, whereas no significant vertical jump increase was observed in the leg-press group. Early increases in strength in untrained individuals are mostly attributable to neural factors, with the contribution of skeletal muscle hypertrophy becoming more important as training continues [12]. Thus, an important consideration when trying to compare these studies is the training status of the subject population [13], which may explain some, but not all, of the mixed results previously reported [8,10,11]. 

As a result of the mixed outcomes when comparing machine-based lower-body training with free-weight squat training, more evidence is needed to validate the superiority, or lack thereof, of resistance training with free weights over machines for sports performance. The purpose of this study was to compare the difference between free-weight squats and machine squats for augmentation of performance in untrained, recreationally active women during an initial resistance training mesocycle. Our experimental hypotheses were that resistance training using free-weight squats would result in a greater improvement in vertical jump height, including calculated peak jump power and average jump power, sprint times, and change-of-direction (COD) times compared with machine squat and control groups. These outcome measures were chosen because of the importance of jumping, maximal speed, and COD in many competitive sports.

## 2. Materials and Methods

### 2.1. Participants

Using G*POWER 3.0 (Universität Düsseldorf, Düsseldorf, Germany) [14], a power analysis determined that 30 participants were needed for a power of 0.80, using a Cohen’s *f* effect size of 0.3 and an α level of 0.05. Initially, 30 women were recruited for the study, but three discontinued participation due to personal reasons or lack of desire to continue. Thus, 27 non-resistance-trained, recreationally active women (age of 22.7 ± 3.5 years; height of 166.61 ± 6.79 cm; body mass of 70.57 ± 16.82 kg) participating in at least 30 min of physical activity weekly completed the study. Exclusion criteria included prior lower-extremity surgery or indication of current or past health conditions, as noted on a health history questionnaire, which may have limited participation in or adaptations to exercise. Non-resistance-trained, recreationally active women were specifically chosen in order to determine the effects of resistance training on athletic performance during an initial training phase. Lack of previous resistance training was confirmed through completion of a physical activity questionnaire. Participants signed an informed consent document approved by the University of South Alabama Institutional Review Board before participation. 

### 2.2. Study Design

This study utilized a block-randomized, parallel design. The type of squat training was used as an independent variable with vertical jump height, sprinting time, and change-of-direction time designated as dependent variables. Participants were block-randomized before the first testing session to one of three groups: free-weight squat (FWS), required to complete all squatting exercises using a free barbell in a safety rack; a machine squat (MS), required to complete all squatting exercises with a hack squat machine (Maxicam, Muscle Dynamics, Paramount, CA, USA), and a control group (CON) that performed no squatting exercises. All groups (FWS, MS, and CON) completed 6 weeks of jumping, sprinting, and COD drills twice weekly. No other exercise training was performed by the participants during the course of the study.

Prior to data collection, participants were familiarized to each performance test over three sessions for a period of one week. After familiarization, subjects completed a pre-testing protocol consisting of one or two sessions, depending on if they were randomized to a squat training group or the control group. During the first pre-testing session, total body mass was assessed (Seca Model 700, Seca Corporation, Chino, CA, USA), followed by performance measures in the following order: vertical jump, pro-agility COD, zig-zag COD, and 30-meter sprint tests. During pre-testing session two, a 1-repetition maximum (1-RM) was determined for the FWS and MS groups, based on group assignment. The control group did not participate in the second pre-testing session. After pre-testing, participants completed a six-week strength training program combined with jump, sprint, and COD training. Post-testing was completed identically to the pre-testing sessions.

#### 2.2.1. Vertical Jump Test

For the vertical jump, participants completed three countermovement jumps with a one-minute recovery period. Participants started by bending their knees, followed by jumping as high as they could while swinging their arms to generate momentum. The Vertec (Perform Better, West Warwick, RI, USA) device was used to determine jump height, from which standing reaching height was subtracted [15]. Peak and average jump power output were determined from the highest vertical jump height and body mass using methods previously described [16].

#### 2.2.2. Change-of-Direction and Sprint Tests 

The pro-agility COD test was performed utilizing standard procedures [17]. The pro-agility COD test started with the participant straddling the start line. The participant started the test by turning to the left and sprinting for five yards, and then the subject turned to the right and sprinted for 10 yards before turning back to the left and sprinting for five yards to the original line, thus marking the end of the test. The Freelap Pro Coach BLE 424 (Pleasanton, CA, USA) timing system was used to record times for the pro-agility COD test. The zig-zag COD test consisted of four five-meter sections marked with cones set at 100 degree angles [18]. Participants performed two trials by running around each cone as fast as possible without losing stability. A two-minute rest was provided between trials. For the 30-meter sprint, participants executed two maximal sprints with a three minute rest between trials. The Speedtrap Timing System (Perform Better Inc., West Warwick, RI, USA) was used to record zig-zag COD and sprint times. The Speedtrap Timing System uses a pressure release mechanism to start the timer. Participants were instructed to place their hand on the pressure sensor using a three-point stance and to begin the test when ready. The participants finished the test by running past an infrared finish-line beam, at which point, the timer was stopped automatically. For all tests, the fastest time was used for datum. 

#### 2.2.3. Squat 1-RM Tests

Participants assigned to the FWS and MS groups only performed the 1-RM test for the squat exercise to which they were assigned, following standard protocols [19]. For each exercise, participants warmed-up by performing six to 10 repetitions, using approximately 50% of their estimated 1-RM. Then, the weight was increased to approximately 70% of estimated 1-RM for a warm-up set of three to five repetitions. After the warm-up sets, weight was added conservatively and the participants were required to perform one repetition. If successful, weight was added until the participant could no longer complete one repetition with proper form. The final weight, lifted successfully and with proper form, was recorded as the absolute 1-RM. Three minutes of rest was allowed between each set. This protocol was used for pre- and post-strength testing for both the FWS and MS groups. All squat exercises were performed to 90 degrees of knee flexion.

#### 2.2.4. Training Sessions

Participants performed two supervised training sessions per week (Table 1). The first session consisted of jumps followed by the squat exercise. For the jumps, participants were coached to perform a counter movement and then jump and reach as high as possible. For the drop jumps, participants were coached to step off a 12-inch box and immediately jump and reach as high as possible upon ground contact. The second session consisted of sprint and COD drills, followed by the squat exercise. All squats were performed to 90 degrees of knee flexion with a cadence of approximately three seconds for the eccentric phase and a concentric phase performed as quickly as possible. The CON group only performed the jumps or drop jumps, sprint, and COD drills. A minimum of 48 h of rest separated each training session.

### 2.3. Statistical Analysis

Performance measures were analyzed using separate 3 × 2 (group by time) two-way mixed-model analyses of variance (ANOVA). If a significant interaction was found, a separate one-way ANOVA was performed to determine any within-group differences over time. Additionally, for significant interactions, a one-way ANOVA was performed using delta scores to determine differences from pre to post between groups. Post-hoc tests were performed using Bonferroni’s test. Within-group differences in maximal strength were analyzed using paired-samples *t* tests. Maximal strength scores were not compared between groups because of the different testing mode (machine vs. free weights). Effect sizes are expressed as partial eta-squared (*η*^2^), with a partial *η*^2^ ≥ 0.02, 0.13, and 0.26 interpreted as small, medium, and large effects, respectively [20]. Intra-class correlation coefficients (ICC) and standard error of the measurement (SEM) were calculated using a two-way mixed effects model for the 30-meter sprint, zig-zag COD, and pro-agility tests since multiple trials were performed. The critical value for significance was set at *p* ≤ 0.05. Analyses were performed using SPSS version 22.0 (IBM, Armonk, NY, USA).

## 3. Results

Outcome data for each variable are found in Table 2. Data for all groups and variables were normally distributed as assessed by Shapiro–Wilk’s test of normality. Maximal strength increased for both the FWS group (*p* < 0.01, partial *η*^2^ = 0.52) and the MS group (*p* < 0.01, partial *η*^2^ = 0.28). Intraclass correlation coefficient values for each performance task were greater than 0.90, indicating excellent reliability. Specifically, ICC values were 0.992, 0.930, and 0.982 for the 30-meter sprint, pro-agility, and zig-zag COD tests, respectively. Additionally, SEM values were 0.04, 0.16, and 0.08 for the 30-meter sprint, pro-agility, and zig-zag COD tests, respectively. At follow-up, a significantly lower 30-meter sprint time (*p* < 0.01, partial *η*^2^ = 0.32, mean change = −0.18 s), pro-agility test time (*p* < 0.01, partial *η*^2^ = 0.49, mean change = −0.55 s), and zig-zag COD time (*p* < 0.01, partial *η*^2^ = 0.58, mean change = −0.31 s) were observed for the main effect of time, with no significant interaction effects (*p* > 0.05).

A significant interaction effect was observed for total body mass (*p* = 0.04, partial *η*^2^ = 0.24). There was an increase in body mass over time for the MS group (*p* = 0.02, partial *η*^2^ = 0.52). A significantly greater increase in total body mass was noted for the MS group compared with CON (*p* = 0.01, partial *η*^2^ = 0.28). A significant interaction effect was observed for peak jump power (*p* = 0.05, partial *η*^2^ = 0.22) and average jump power (*p* = 0.03, partial *η*^2^ = 0.27). A significant increase in peak jump power over time was observed for the CON (*p* = 0.03, partial *η*^2^ = 0.49) and MS (*p* < 0.01, partial *η*^2^= 0.64) groups, but not the FWS group (*p* > 0.05). Group comparisons revealed the change in peak jump power to be greater for the MS group compared with the FWS group (*p* = 0.05; partial *η*^2^= 0.22). A significant increase in average jump power over time was revealed for the MS group (*p* < 0.01, partial *η*^2^ = 0.81), but not the FWS or CON groups (p > 0.05). Additionally, change in average jump power was greater for the MS group compared with the CON group (*p* = 0.04, partial *η*^2^ = 0.47) but not the FWS group (*p* = 0.09, partial *η*^2^ = 0.28). A significant increase in vertical jump height was observed for the main effect of time (*p* < 0.01, partial *η*^2^ = 0.41), with no significant interaction effect (*p* = 0.08). 

## 4. Discussion

The main finding of this study was that the average and peak jump power for the MS group increased to a greater degree than the CON and FWS groups, respectively. Neither the free-weight nor machine squat training contributed any additional benefit to jump height, sprint, or COD performance, suggesting the initial improvements in jump height, sprint, and COD in untrained women are a result of direct training and not from increases in maximal strength. Further, the lack of influence does not appear to result from a lack of training-induced strength gains, as maximal strength increased significantly in both groups. 

Regarding jumping ability, our results offer similarities and differences with those of previous studies. Silvester and Bryce [8] reported similar increases in vertical jump height for machine and free-weight training. We also found similar increases in jump height between MS and FWS training; however, the increases in jump height were no greater than the CON group performing just the performance tasks. In this population, it appears that increases in vertical jump height can be obtained with minimal training. Further, it may be that both resistance training and sport-specific training can increase vertical jump, but that they do not offer an additive effect as the maximal rate of adaptation may be reached by resistance training or sport-specific training alone. Interestingly, our results indicated a significant increase in total body mass only for the MS group. Despite this increase, the MS group was still able to increase vertical jump height to the same degree as the FWS and CON group, leading to preferential increases in jumping power for the MS group. We find it plausible that the MS allowed for focus to be placed on force output during the training sessions, whereas more attention to technique was needed with FWS training. Thus, in our population of recreationally active women, MS allowed for quicker adaptations regarding lower-body power output. Greater emphasis on force output rather than technique could have contributed to slightly more hypertrophy in the MS group, explaining the increase in body mass not seen in the other two groups. However, this should be interpreted with caution as neither body composition nor force output were measured during this study. Future studies should determine if MS training in untrained women results in greater jumping power, measured via a force plate device, after an initial training mesocycle compared with FWS training.

It should be noted that others have reported machine-based squat training to be ineffective in eliciting improved jumping performance when compared with subjects completing no training [10]. Hence, more research is needed to explore the factors determining the transfer of machine training to performance outcomes. In doing so, it is also important to consider the type of machine training employed when considering free-weight training versus machine training. The current study utilized a hack squat machine, which is a closed-chain movement like the barbell squat. When comparing free weights and machines, Rossi et al. [9] and Wirth et al. [11] utilized leg-press machine training, an open-chain exercise, with differing outcomes. Rossi et al. [9] observed no statistical difference between leg-press training and free-weight training on vertical jump height, whereas, Wirth et al. [11] reported that free-weight squats increased vertical jump performance in athletes, with no similar improvement observed as a result of leg-press training. Thus, in addition to population type, the type of machine exercise employed could further affect training outcomes. 

Our results may reflect a specific outcome as a result of our sample population being untrained as two prior studies reported improved sprint running velocities following squat training in soccer players [4,5]. Additionally, increases in maximal squat strength were associated with improved sprint performances in rugby league players [6]. At the very least, our results indicate the importance of task-specific training in addition to resistance training. In agreement, another study reported that plyometric training without resistance training improved times on COD tests compared to a control group, although the training status of the participants was not clearly defined [21]. A meta-analysis by Seitz et al. [22] indicated that there is a transfer between increases in lower-body strength and sprint performance. The authors also indicated that numerous other factors affect sprint performance, one of which is level of practice. We observed improved performance in jump height and COD and sprint times as a result of task-specific practice in the control group. Thus, for untrained women, practice of the specific jump, sprint, or COD tasks may be most important for improving performance initially, with increases in lower-body maximal strength becoming more important as the athlete becomes more well-skilled. Importantly, our data suggest a minimal amount of training volume is needed to increase maximal lower body strength in untrained women. Minimum effective training volume for increasing maximal strength in untrained women may be as little as 3 to 6 sets performed two times per week for 6 weeks.

Our study contains limitations that should be addressed with future research. Notably, to strengthen the comparison between groups, 1-RM measures should have been obtained for each exercise and time point for all three groups. The absence of this data limits more comprehensive interpretation of the results and disallowed comparison of baseline strength. Further to this point, the lack of data pertaining to external load and/or internal load (e.g., rating of perceived exertion) should be considered a limitation. Another limitation is that jumping power output was based on calculations using vertical jump height and body mass, as such, these measurements are prone to error. Lastly, because our results are in untrained women, it is important to emphasize these results may not be applicable to elite athletes or men. Nonetheless, this study is important because there is a considerable gap in the literature regarding the systematic study of how different resistance exercise training modalities contribute to sports-specific performance. This disparity is further magnified when considering differences in age, sex, training status, and type and level of sport participation.

## 5. Conclusions

In conclusion, our overall findings do not support our hypothesis that squat training using free weights is superior to machine resistance for the improvement of jump height, speed, and agility over an initial six-week period in untrained, recreationally active women. Conversely, machine squats training resulted in greater jumping power compared with free-weight squat training. Our results indicate MS training is at least equivalent to FWS training for increasing jump height, speed, and COD ability. Machine squat training may be a suitable initial training modality for previously untrained women with progression to FWS after proper technique is mastered, and may even be superior to FWS for increasing jumping power during initial phase training. For speed and COD, compared to solely training the sport-specific tasks, six weeks of resistance training in a previously untrained population of healthy women did not appear to further augment performance. Thus, specific training of vertical jump, speed, and COD appears to be the most important factor for immediate improvement in these variables in this population. It is imperative to note that our findings are only applicable to previously untrained women, and it is likely that training adaptations would differ in moderately to well-trained women. Future research should directly explore the magnitude of the potential differential effects of MS training and FWS training on performance in trained and untrained individuals. Highly trained individuals may be better at transferring newly acquired strength to performance tasks, although this needs to be studied directly. 

## Figures and Tables

**Table 1:** Outline of training sessions.

Week	Sessions 1 and 2			Session 1				Session 2		
	Squat			Jumps		Drop Jumps		Sprint	Pro-Agility	Zig-Zag
	Sets	Reps	Rest	Sets	Reps	Sets	Reps	Sets	Sets	Sets
1	3	10–12	90 s	2	5	-	-	2 *	2 *	2 *
2	3	8–10	90 s	3	5	-	-	2 *	2 *	2 *
3	4	6–8	90 s	3	5	-	-	2 **	2 **	2 **
4	5	6–8	90 s	-	-	3	5	3 *	3 *	3 *
5	5	3–5	120 s	-	-	3	5	3 *	3 *	3 *
6	6	3–5	120 s	-	-	4	5	4 **	4 **	4 **

*Note*. * performed at 90% of subjective peak effort; ** last set performed using peak effort. For Week 1, the squat load was set at ~70% of 1-RM. Load was increased conservatively if the participant was able to complete more than the required amount of repetitions on the set performed. Interindividual progressions were variable. Typically, the amount of weight added decreased the maximum amount of repetitions able to be performed to within the middle of the prescribed repetition range.

**Table 2:** Mean and Standard Deviation (*M ± SD*) of outcome variables for each group and time point.

Variable	Time	CON	FWS	MS	TOTAL
Age (years)		23.89 ± 3.72	22.33 ± 3.20	21.89 ± 3.59	22.70 ± 3.48
Height (cm)		165.28 ± 4.70	164.56 ± 7.80	170.00 ± 6.88	166.61 ± 6.79
Body Mass (kg) ^††^	Pre	79.24 ± 21.87	64.68 ± 13.44	67.79 ± 11.35	70.57 ± 16.82
	Post	79.00 ± 22.49	65.39 ± 12.78	69.27 ± 11.06 *	71.22 ± 16.66
	Δ	0.24 ± 1.61	0.71 ± 1.70	1.47 ± 1.03 ^C^	0.64 ± 1.59
Squat 1-RM (kg)	Pre	-	50.76 ± 9.09	77.02 ± 19.53	-
	Post	-	69.09 ± 8.81 **	100.76 ± 18.07 **	-
	Δ	-	18.43 ± 6.86	23.74 ± 7.20	-
Vertical Jump (cm) ^†^	Pre	37.25 ± 5.99	33.87 ± 5.43	36.55 ± 8.82	35.89 ± 6.80
	Post	39.79 ± 6.75	34.85 ± 6.60	41.49 ± 10.32	38.71 ± 8.27 **
	Δ	2.54 ± 2.77	0.98 ± 2.89	4.94 ± 4.73	2.82 ± 3.82
Peak Jump Power (W) ^††^	Pre	6981 ± 765	6247 ± 555	6525 ± 339	6584 ± 637
	Post	7129 ± 713 *	6333 ± 583	6884 ± 514 **	6782 ± 676
	Δ	148 ± 162	86 ± 228	359 ± 283 ^F^	198 ± 251
Avg. Jump Power (W) ^††^	Pre	3526 ± 491	3163 ± 307	3257 ± 209	3316 ± 376
	Post	3542 ± 493	3188 ± 293	3332 ± 215 **	3354 ± 371
	Δ	16 ± 37	25 ± 59	75 ± 39 ^C^	38 ± 52
30-Meter Sprint (s) ^†^	Pre	5.87 ± 0.61	5.75 ± 0.36	5.73 ± 0.67	5.79 ± 0.55
	Post	5.75 ± 0.51	5.67 ± 0.34	5.43 ± 0.62	5.61 ± 0.50 **
	Δ	−0.12 ± 0.25	−0.08 ± 0.26	−0.30 ± 0.29	−0.17 ± 0.27
Zig-Zig Test (s) ^†^	Pre	7.07 ± 0.59	7.29 ± 0.46	6.86 ± 0.57	7.07 ± 0.55
	Post	6.76 ± 0.62	6.95 ± 0.44	6.55 ± 0.74	6.76 ± 0.61 **
	Δ	−0.30 ± 0.27	−0.33 ± 0.26	−0.31 ± 0.32	−0.31 ± 0.29
Pro-Agility Test (s) ^†^	Pre	6.17 ± 0.60	6.76 ± 0.85	6.61 ± 1.04	6.51 ± 0.86
	Post	5.91 ± 0.49	6.18 ± 0.46	5.79 ± 0.66	5.96 ± 0.55 **
	Δ	−0.26 ± 0.35	−0.58 ± 0.82	−0.82 ± 0.52	−0.55 ± 0.62

*Note*. CON = control group; FWS = free-weight squat group; MS = machine squat group. ^†^ indicates significant main effect for time; ^††^ indicates significant group by time interaction effect; * indicates significantly different from pre (*p* < 0.05); ** indicates significantly different from pre (*p* < 0.01); ^C^ indicates significantly different from Control Group; ^F^ indicates significantly different from Free-Weight Squat Group.
